# The Kinase ERULUS Controls Pollen Tube Targeting and Growth in *Arabidopsis thaliana*

**DOI:** 10.3389/fpls.2017.01942

**Published:** 2017-11-14

**Authors:** Sébastjen Schoenaers, Daria Balcerowicz, Alex Costa, Kris Vissenberg

**Affiliations:** ^1^Integrated Molecular Plant Physiology Research, University of Antwerp, Antwerp, Belgium; ^2^Department of Biosciences, University of Milan, Milan, Italy; ^3^Institute of Biophysics, Consiglio Nazionale delle Ricerche, Milan, Italy; ^4^Plant Biochemistry and Biotechnology Lab, Technological Educational Institute of Crete: University of Applied Sciences, Crete, Greece

**Keywords:** ERULUS, calcium, fertilization, kinase, pollen tube, tip growth

## Abstract

In this paper, we describe the role of the receptor-like kinase ERULUS (ERU) in PT growth of *Arabidopsis thaliana*. *In silico* analysis and transcriptional reporter lines revealed that *ERU* is only expressed in pollen and root hairs (RHs), making it a tip growth-specific kinase. Deviations from Mendelian inheritance were observed in the offspring of self-pollinated heterozygous *eru* plants. We found that *in vivo eru* PT targeting was disturbed, providing a possible explanation for the observed decrease in *eru* fertilization competitiveness. Extracellular calcium perception and intracellular calcium dynamics lie at the basis of *in vivo* pollen tube (PT) tip growth and guidance. *In vitro, ERU* loss-of-function lines displayed no obvious PT phenotype, unless grown on low extracellular calcium ([Ca^2+^]_ext_) medium. When grown at 12 the normal [Ca^2+^]_ext_, *eru* PTs grew 37% slower relative to WT PTs. Visualization of cytoplasmic [Ca^2+^]_cyt_ oscillations using the Yellow Cameleon 3.6 (YC3.6) calcium sensor showed that, unlike in WT PTs, *eru* apical [Ca^2+^]_cyt_ oscillations occur at a lower frequency when grown at lower [Ca^2+^]_ext_, consistent with the observed reduced growth velocity. Our results show that the tip growth-specific kinase ERULUS is involved in regulating Ca^2+^-dependent PT growth, and most importantly, fertilization efficiency through successful PT targeting to the ovules.

## Introduction

Pollen tube (PT) tip growth is a highly polarized form of cell elongation ultimately leading to the delivery of sperm cells at the embryo sac and subsequent double fertilization (Obermeyer and Feijó, 2017). The PT tip functions as the site of plasticity and extension, where vesicles filled with cell wall proteins and precursors fuse with the apical plasma membrane, providing extra membrane and cell wall material to the growing cell. Simultaneously, the PT apex provides the interface at which extracellular cues are perceived and translated toward changes in PT growth directionality.

During its penetration down the style and transmitting tract toward the ovule, many interactive signals are exchanged between the PT and its surrounding tissue (Qu et al., 2015; Obermeyer and Feijó, 2017). Upon reaching the female gametophyte, the PT encounters attracting signals that guide it to the synergids. These synergids, which flank the egg cell, secrete small cysteine-rich proteins (LUREs) that probably act as signaling ligands (Okuda et al., 2009; Takeuchi and Higashiyama, 2016). LUREs attract the incoming PT toward the target egg cell, a process that involves the membrane bound RLKs LIP1 and LIP2 (Liu et al., 2013). Subsequently, on the synergid cells, the receptor-like kinase (RLKs) FERONIA/SIRENE and the GPI-anchor protein LORELEI interact with an unknown signal, finally causing the cessation of PT growth (Miyazaki et al., 2009; Liu et al., 2016). ZmES4 signals to the PT tip and induces bursting, which involves activation of PT plasma membrane-localized KZM1, a K^+^ Shaker channel (Amien et al., 2010). Sperm cell discharge is controlled by RLKs ANXUR1/2 and the activity of a Ca^2+^ transporter, ACA9, that are all expressed in the PT (Schiott et al., 2004; Miyazaki et al., 2009).

The ability of PTs to perceive their extracellular environment and respond adequately by adjusting their growth regime seems to coincide with specific intracellular [Ca^2+^] signatures (Iwano et al., 2012; Damineli et al., 2017). More so, Ca^2+^-signatures seem embedded throughout the male–female tissue crosstalk pathway (Denninger et al., 2014; Hamamura et al., 2014). A number of highly dynamic cellular processes occur during PT growth (Obermeyer and Feijó, 2017), including the formation and maintenance of intra- and extracellular ion dynamics, apical endo-/exocytosis and the modification of the cytoskeleton, all of which depend on tight spatial and temporal control of cytosolic [Ca^2+^] oscillations at the tip (Schoenaers et al., 2017). Pharmacological interruption of the apical cytoplasmic Ca^2+^ gradient causes immediate growth cessation (Iwano et al., 2009), and local elevation of the [Ca^2+^]_ext_ irreversibly steers tip growth directionality *in vitro* (Bibikova et al., 1997). *In vivo* alteration of the [Ca^2+^]_ext_ might be an important factor in controling PT guidance. For instance, the [Ca^2+^]_ext_ was found to increase in the Lily transmitting tract upon pollination (Zhao et al., 2004). More so, pollination induces transcription of the Ca^2+^ export system *AUTOINHIBITED CALCIUM-ATPASE 13* (*ACA13*) in the *Arabidopsis* transmitting tract (Iwano et al., 2014). Most importantly, Ca^2+^ facilitates pectin cross-linking and ROS production in the cell wall (Rounds et al., 2011; Mangano et al., 2016). As such, Ca^2+^ ions have a pivotal role in controling cell wall flexibility, a *sine qua non* condition for (oscillatory) PT elongation.

Despite these findings, it remains poorly understood how small changes in the [Ca^2+^]_cyt_ oscillatory regime are regulated, and how they can lead to alterations of PT growth. In addition, despite our improved understanding of the process of PT guidance and fertilization, the number of molecular players that are known to be involved is limited. With the characterization of FER and ANXUR1/2 and their role in Ca^2+^ mediated fertilization, members of the CrRLK1L family have gained attention to the study of polarized growth and its regulation. ERULUS (ERU), also a CrRLK1L protein, has been described as a core root hair (RH) regulator, involved in the establishment of a functional apical [Ca^2+^]_cyt_ gradient (Bai et al., 2014a). *ERU* loss-of-function RHs are short and stunted and apparently have a perturbed tip [Ca^2+^]_cyt_ accumulation. Here we describe a role for ERU during fertilization. We found that *ERU* is a PT-expressed kinase that is involved in Ca^2+^-dependent PT growth and the control of *in vivo* PT guidance and fertilization.

## Materials and Methods

### Plant Material and Growth Conditions of *Arabidopsis*

*Arabidopsis thaliana* ecotype Columbia-0 (Col-0) and mutant *eru* (SALK_083442C) seeds were obtained from the Nottingham *Arabidopsis* stock center. Plants homozygous for the *eru* T-DNA insert were selected by PCR using T-DNA and gene-specific primers (Supplementary Table 1), backcrossed to the Col-0 background twice and reselected for the *eru* T-DNA insert.

WT and *eru* plants were grown in soil (Tref substrate) and kept in a growth room at 21°C in a 16/8 h light period under a light intensity of 70–90 μmol m^-2^s^-1^.

Pollen from stage 13–15 flowers were germinated in the dark at 21–23°C in a closed Petri dish on a thin cellophane membrane overlaying solid pollen growth medium in accordance with Rodriguez-Enriquez et al. (2012). The medium was supplemented with 0.5, 1, or 2 mM CaCl_2_ or 1 mM (NH_4_)H_2_PO_4_ when appropriate. The solution was heated in a microwave for the agarose to dissolve and cooled down to 50–60°C for the pH to be readjusted. A 0.5 cm × 0.5 cm cellophane membrane (325P cellulose; AA Packaging Limited, Preston, United Kingdom) was placed on top of the medium, and pollen were applied directly onto the membrane.

### *In Silico* Analysis

The putative 1500 bp promoter region of ERULUS was examined for cis-elements using Place^1^. Public transcriptomics data was consulted using the eFP browser (Winter et al., 2007) and Genevestigator (Hruz et al., 2008).

### Molecular Cloning and Plant Transformation

Constructs were generated using the Gateway Cloning system^2^ (Life Technologies). Genomic DNA was extracted from WT seedlings using phenol extraction. For the promoter::reporter gene analysis a 619bp sequence upstream of the start codon of *ERULUS* was PCR amplified from Col-0 genomic DNA using Platinum high fidelity DNA polymerase (Life Technologies). The following primers were used: 5′-GGGGACAAGTTTGTACAAAAAAGCAGGCTTC-GCTTTGAGGTCATTTTT-3′ and 5′-GGGGACCACTTTGTACAAGAAAGCTGGGTAATATCCGGCGAGGTTTTG-A-3′. PCR products were subcloned into pDONR207 and the sequence was verified by DNA sequencing. The promoter region was subsequently fused to the β-glucuronidase reporter using the LR reaction and the pGWB3 destination vector (Nakagawa et al., 2007). The construct was electroporated into *Agrobacterium tumefaciens* C58. Plant transformation was done by the flower dip method (Clough and Bent, 1998). Seeds were selected on half strength MS medium containing 50 μg ml^-1^ Kanamycin or 25 μg ml^-1^ Hygromycin B. Homozygous T_3_ progeny was used for further analysis. Eight independent homozygous lines were examined for GUS expression.

### GUS Staining

GUS activity staining was performed in WT plants homozygous for the *proERU::GUS* insert according to a modified protocol of Jefferson et al. (1987).

### Pollen Germination and PT Growth Dynamics

To determine pollen germination, *eru* and Col-0 pollen from stage 15 flowers were grown on pollen medium and the percentage of germinated pollen was counted 16 and 24 h after pollination.

To quantify PT growth dynamics, *eru* and Col-0 pollen were grown on pollen medium and gently overlaid by a cover glass directly onto the medium. Time-lapse movies were collected for approximately 1 h with a framerate of 20 s using transmitted light on a Zeiss Axioplan microscope using a Zeiss Achroplan 100× (na 1.25) oil immersion Ph3 objective. The PT length gain was measured for each frame. The data for individual PTs was aligned based on their initial length, averaged, and plotted against time.

### Fertilization Competitiveness

Siliques of the T_3_ progeny derived from three self-pollinated heterozygous *eru* T_2_ plants were investigated. Fifteen siliques from each plant were collected and cut in the middle to generate a top half and a bottom half as described previously (Schiefelbein et al., 1993). Due to the fact that the T-DNA-specific Kanamycin resistance gene was found to be silenced in the *eru* mutant and that this mutation is recessive, seeds were sown on solid RH medium and the percentage of mutants was calculated by visual screening of the obvious *eru* RH phenotype (Bai et al., 2014a; Haruta et al., 2014).

### Aniline Blue Staining of *in Vivo* Grown PT

Stage 12 WT flowers were emasculated and stigma were hand-pollinated with WT or *eru* pollen from stage 13–15 flowers. Twenty-four hours after pollination, the pistils were fixed for 2 h in 1:3 acetic acid:ethanol solution, softened overnight in 8 M NaOH, cleared with distilled water and subsequently stained with decolorized aniline blue (0.1% filtered aniline blue in 0.1 M K_2_HPO_4_-KOH buffer, pH 11) for 2 h in the dark (Mori et al., 2006). PTs were subsequently visualized by exciting DAB-stained callose using UV lighting on a Zeiss Axioplan fluorescence microscope. More than 300 PTs originating from 10 (*eru* pollinated) or 13 (WT pollinated) ovaries were classified according to their targeting phenotype. The length of these PTs was quantified as the distance between the stigma’s surface and the tip of the PT.

### Calcium Imaging

*Agrobacterium* GV3101 containing the NES-YC3.6 harboring pTKan vector with UBQ10 promoter (Krebs et al., 2012) was used to transform *eru* and WT plants by the floral dip method (Clough and Bent, 1998). Positive transformants were selected on MS plates containing 50 mg L^-1^ Kanamycin and transferred to soil. Their progeny was screened for fluorescence using a Nikon AZ100 macroscope coupled to a fluorescence unit, and seeds were collected from plants showing a clear cpVenus-based fluorescence. Note that throughout the text, for simplicity, we will use YFP when referring to cpVenus.

Pollen from plants grown in long day cycles were used for subsequent *in vitro* germination and calcium imaging. A setup was devised to visualize growing PTs on an inverted Nikon Eclipse Ti-E. A small cover glass was covered by a thin film of pollen growth medium (prepared fresh each day), which was overlaid with a 0.5 cm × 0.5 cm square of cellophane membrane. Agarose was chosen as a gelling agent for its lack of ‘contaminating’ calcium compared to other gelling agents, and the fact that it does not rely on the presence of bivalent cations for matrix establishment. In addition, this specific solid medium was used to more closely resemble *Arabidopsis* dry stigma conditions and it was confirmed that its use leads to *in vivo*-like pollen germination and PT growth (Rodriguez-Enriquez et al., 2012). Finally, the use of solid medium prevented sample drifting during prolonged imaging. Pollen were applied on the membrane and the cover glass was flipped on another cover glass that was attached to an opening at the bottom of a small Petri dish. Water was applied on the inner sides of the Petri dish to maintain high humidity and the Petri dish was closed using parafilm. The pollen grains were germinated in a climate controlled room at 23°C, in the dark, and visualized approximately 2 h after pollination.

YC3.6 positive PTs were visualized at the tip using a 60× oil immersion objective and excited using the Prior Lumen 200 PRO fluorescent lamp (Prior Scientific) at 440 nm (436/20 nm). Images were collected with a Hamamatsu Dual CCD Camera ORCA-D2. CFP (465–500 nm) and FRET-induced YFP (520–570 nm) emission were detected simultaneously every 5 s with an exposure time of 300–400 ms using a beam splitter (A11400-03 optical block; 483/32 nm for CFP; 542/27 nm for YFP) and Hamamatsu dichroic 510 nm mirror. Images were collected simultaneously for each channel using binning 4 × 4.

Image analysis was performed in Fiji ImageJ (Schneider et al., 2012). For both channels, all frames were aligned with respect to the PT tip using the stackreg plugin and the rigid body detection algorithm. The YFP and CFP fluorescence intensities were extracted from the very tip of the growing PT, as well as the background signal intensity. The background intensity value was subtracted independently from both YFP and CFP gray values before YFP/CFP ratio calculation. The change in the ratio (ΔR) was normalized to the average ratio of the series (<R>) and plotted versus time (ΔR/<R>). The Ratio Plus Plugin for Fiji was used to visualize the calcium oscillations. Oscillograms were analyzed by Fourier transformation, in order to isolate the main oscillation frequency.

### Statistics

Statistics were performed using the R statistics platform (R Core Team, 2008). Significance (α = 0.05) was assessed by two-way analysis of variance (ANOVA; parametric) using linear mixed-effects models followed by a TukeyHSD (for pairwise statistical analysis), or Kruskal–Wallis tests (non-parametric).

## Results

### *ERULUS* Is Transcribed in Mature Pollen Grains and PTs

**FIGURE 1:**
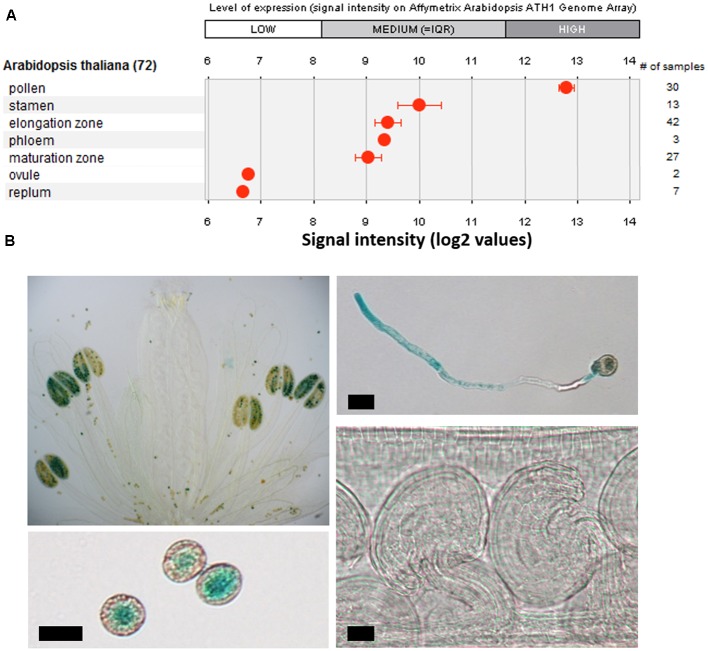
Characterization of tissue specific *ERU* transcription. **(A)** Tissue specific expression of *ERU* based on publicly available transcriptomics datasets (Genevestigator). **(B)** GUS-staining of WT × *promERU::GUS* flowers, pollen, pollen tubes (PTs), and ovules. Scale bars = 10 μm.

Recent data showed that *ERULUS*, a RLK from the *Arabidopsis thaliana* subfamily of *Catharanthus roseus* RECEPTOR-LIKE KINASE 1-LIKE proteins (CrRLK1Ls), is expressed in trichoblast cells and that it controls tip growth of RHs in *Arabidopsis* (Bai et al., 2014a; Haruta et al., 2014). In higher plants, tip growth is restricted to RHs and PTs. Publicly available micro-array data indicated that *ERULUS* is transcribed in pollen too (**Figure 1A**). We performed an *in silico* analysis of the 1500 bp upstream promoter region and found that the *ERU* promoter contains two auxin response elements (AuxREs; TGTCTC), putative binding sites for auxin response factors (ARFs) which regulate auxin-induced transcription (Ulmasov et al., 1997), and (in addition to two RH-specific *cis*-elements), six pollen-specific *cis*-elements (Supplementary Table 1).

We visualized *ERU* expression by GUS staining in stably transformed WT x *proERU::GUS* plants and found that, in the areal parts, *ERU* transcription is restricted to mature pollen grains and *in vitro* growing PTs (**Figure 1B**). GUS staining was not detected in any other flower part, even after prolonged staining.

### Standard Growth Conditions Do Not Affect *eru* Pollen Development

Given the apparent tip growth phenotype of *eru* RHs and the possibility of a similar defect in growing PTs, we performed an *in vitro* phenotyping of *eru* pollen and PTs. We compared pollen viability by Alexander staining and found no difference in viability between WT and *eru* mature pollen grains (Supplementary Figure 1A). Next, we germinated mutant pollen under standard growth conditions. Both WT and *eru* pollen germinated and produced normal looking PTs (Supplementary Figure 1B). No significant difference between *eru* and WT was detected with respect to both pollen germination (Supplementary Figure 1C) and PT length (Supplementary Figure 1D). In addition, callose plug formation, a prerequisite for normal sperm migration in the PT (Qin et al., 2012), was unaffected in *eru* PTs (Supplementary Figure 1E). Bai et al. (2014a) suggested that ERULUS plays a role in ammonium homeostasis in RHs. We therefore investigated the effect of ammonium supplementation on *in vitro eru* and WT pollen germination and PT growth. NH_4_^+^ was supplemented to the medium and the pollen germination percentage (Supplementary Figures 2A,B) and PT length (Supplementary Figures 2A,C) were quantified 2.5 h after pollen application, when PTs are still in the process of growing. We observed no significant difference between WT and *eru* pollen in both control and NH_4_^+^ supplemented conditions, implying that excess NH_4_^+^ does not affect pollen germination (Supplementary Figure 2B), PT length (Supplementary Figure 2C), and PT morphology (Supplementary Figure 2A).

### *eru* PT Growth Is Altered When Grown under Low [Ca^2+^]_ext_

**FIGURE 2:**
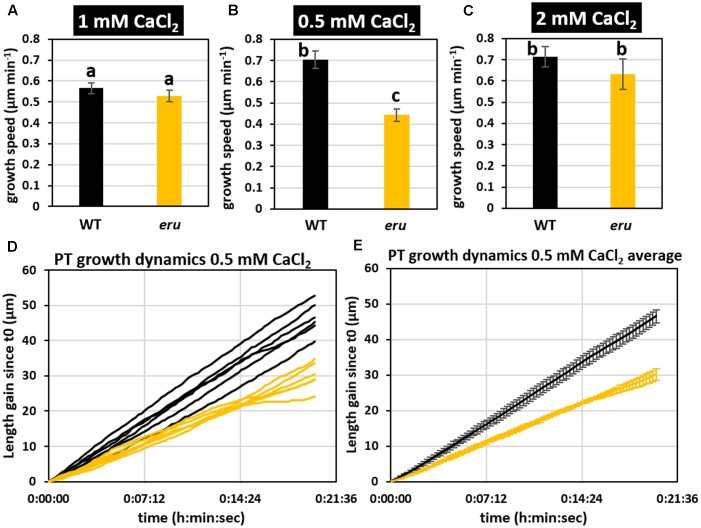
Wild type and *eru* PT growth rates differ in low [Ca^2+^]_ext_ conditions. **(A)** WT and *eru* PT growth rate on standard (1 mM CaCl_2_), **(B)** low calcium (0.5 mM CaCl_2_), and **(C)** high calcium (2 mM CaCl_2_) growth medium. **(D)** PT growth dynamics of WT and *eru* PTs grown in low calcium (0.5 mM CaCl_2_) conditions. Individual traces are represented by black (WT) and orange (*eru*) lines. **(E)** Average of the PT growth dynamics presented in **(D)**. Error bars represent SE. Different letters reflect a pairwise significant difference (α ≤ 0.05).

Given the importance of calcium signaling in PT growth and the role of ERU in establishing a functional calcium gradient during RH tip growth (Bai et al., 2014a), we grew mutant pollen on solid medium containing 1 mM (control), 0.5 mM (low) or 2 mM CaCl_2_ (high). In agreement with our previous observations (Supplementary Figure 1) we observed no significant differences with respect to average *eru* and WT PT growth velocities when grown under standard conditions (**Figure 2A**). Strikingly though, in response to 0.5 mM CaCl_2_ WT PTs grew faster than in the control condition (**Figure 2B**; 0.70 ± 0.04 vs. 0.57 ± 0.02 μm min^-1^) whereas *eru* PTs grew slower on lower [Ca^2+^]_ext_ (0.44 ± 0.03 vs. 0.70 ± 0.04 μm min^-1^). As a result, *eru* PTs grew 37% slower compared to WT PTs at low [Ca^2+^]_ext_ conditions. When grown on medium with increased [Ca^2+^]_ext_ (2 mM CaCl_2_), both WT and *eru* PTs grew faster than in control conditions but no relative difference in average growth rate was seen (**Figure 2C**). Twenty minutes tracking of individual PT growth confirmed a consistently lower growth rate throughout the acquisition time (**Figures 2D,E**). Together, these findings indicate that ERU has a role in regulating PT growth velocity in response to [Ca^2+^]_ext_.

### *ERU* Loss-Of-Function Alters Tip-Focused Calcium Oscillations in Growing PTs in Response to [Ca^2+^]_ext_

**FIGURE 3:**
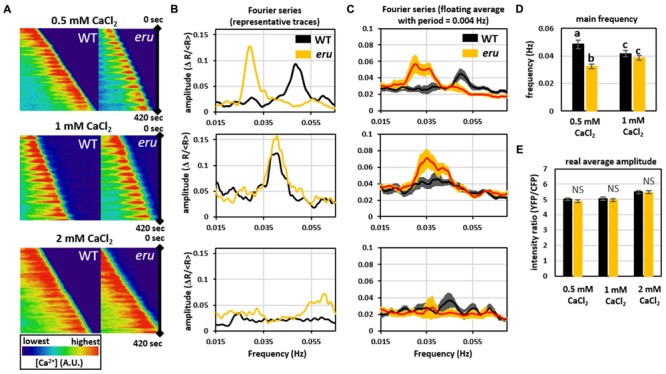
Analysis of [Ca^2+^]_cyt_ oscillations at the tip of growing WT and *eru* PTs at different [Ca^2+^]_ext_. **(A)** Kymograph of 420 sec acquisitions showing regular [Ca^2+^]_cyt_ oscillations at the tip of growing WT and mutant PTs when grown on medium containing 0.5 mM or 1 mM CaCl_2_. **(B)** Representative Fourier transform series of apical calcium dynamics in WT and *eru* PTs. Distinct main frequency peaks were observed at 0.5 and 1 mM, but not at 2 mM CaCl_2_. **(C)** Graphs showing the average of all acquired Fourier transform series. Curves were smoothed using a floating average with a period of 0.004 Hz. **(D)** Bar plot showing the main frequency of oscillation. Main Fourier frequency peaks were isolated from individual Fourier traces and averaged. Fourier analysis did not detect frequency peaks in PTs grown on 2 mM CaCl_2_. **(E)** Average [Ca^2+^]_cyt_ signal amplitude. Error bars represent SE. Different letters reflect a pairwise significant difference (α ≤ 0.05).

To further dissect ERU-mediated signal transduction in response to [Ca^2+^]_ext_, we visualized the tip-focused [Ca^2+^]_cyt_ gradient in growing *eru* and WT PTs. The cytosol-targeted Yellow Cameleon 3.6 (NES-YC3.6) calcium sensor was introduced in *eru* and WT plants by *Agrobacterium*-mediated transformation and [Ca^2+^]_cyt_ oscillations were visualized in *in vitro* growing PTs under low (0.5 mM), normal (1 mM), and high (2 mM) [Ca^2+^]_ext_ (**Figure 3**). Kymograms of the recorded cytoplasmic calcium dynamics indicated that in all conditions oscillating tip-focused calcium gradients were present (**Figure 3A**). Fourier analysis of the acquired oscillograms revealed that WT and *eru* PTs showed similar oscillation frequencies (0.042 ± 0.002 and 0.039 ± 0.002 Hz, respectively) and amplitude (5.05 ± 0.12 and 4.96 ± 0.10, respectively) under control conditions (**Figures 3B–E**). In the presence of high [Ca^2+^]_ext_ (2 mM CaCl_2_), oscillations occurred so rapidly that image acquisition every 5 sec could not resolve separate peaks (**Figure 3A** at the bottom). However, the average amplitude (5.48 ± 0.10 for WT PTs and 5.46 ± 0.10 for *eru* PTs) remained unaffected (**Figure 3E**). Strikingly, however, WT PTs responded to low [Ca^2+^]_ext_ (0.5 mM CaCl_2_) by increasing their main oscillation frequency from 0.042 ± 0.002 to 0.048 ± 0.003 Hz (**Figures 3B–D**). Contrastingly, and in direct agreement with the average growth speed response, the main oscillation frequency in *eru* PTs decreased to 0.033 ± 0.002 Hz when subjected to low [Ca^2+^]_ext_ (**Figures 3B–D**). Together, these results show that ERU is involved in regulating apical [Ca^2+^]_cyt_ oscillations in response to [Ca^2+^]_ext_.

### The *eru* Loss-Of-Function Mutation Leads to Aberrant PT Targeting and Decreased Fertilization Competitiveness

**FIGURE 4:**
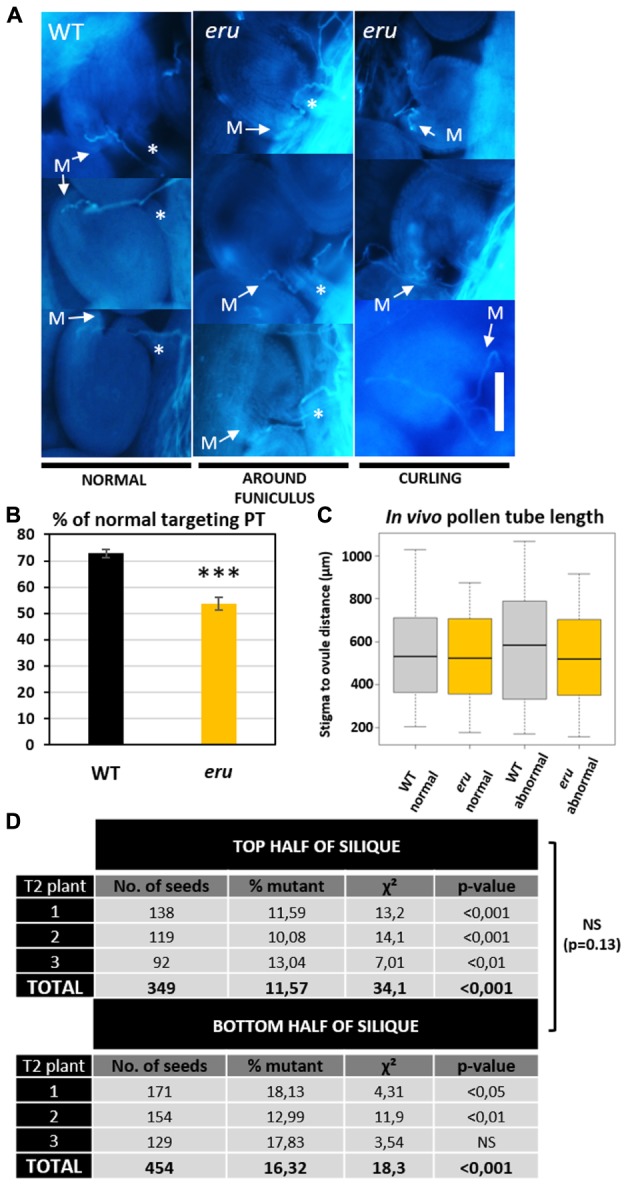
*In Vivo* PT targeting and fertilization efficiency of WT and *eru* pollen. **(A)** Classification of *in vivo* grown pollen tubes using aniline blue staining. More than 300 PTs originating from 10 (*eru* pollinated) or 13 (WT pollinated) ovaries were classified. PTs either grow straight toward the micropyle (M) or show abnormal targeting: turning around the funiculus (marked by ^∗^) or curling. Scale bars = 30 μm. **(B)** Quantification of the PT targeting phenotype. **(C)** Quantification of *in vivo* PT length for normal and abnormal targeted WT and *eru* PTs. **(D)** Result of heterozygous *eru*-plant self-pollination. Homozygous offspring was scored in the top and bottom half of the resulting siliques for three plants. Significance codes *p*-value: ^∗^*p* = 0.05; ^∗∗^*p* = 0.01; ^∗∗∗^*p* < 0.001.

Given the role of calcium-mediated cross-talk during the PTs journey through the transmitting tract toward the ovule, we investigated *in vivo* PT targeting of WT and *eru* pollen. We emasculated non-pollinated WT flowers, hand-pollinated them with WT or *eru* pollen and visualized *in vivo* grown PTs using aniline blue staining. *ERU* loss-of-function PTs grew through the WT transmitting tract in a WT-like manner. However, compared to WT PTs, *eru* PTs were more likely to show aberrant ovule targeting (**Figures 4A,B**). More specifically, proportionally more mutant PTs grew around the funiculus or made multiple turns before targeting the micropyle (**Figure 4A**). Defective targeting was independent of the PT length (**Figure 4C**). Hence, aberrant targeting was not skewed toward ovules that were either situated at the top or bottom of the ovary.

To understand the biological relevance of this phenotype, we quantified the transmission efficiency of the *eru* mutant allele versus that of the WT *ERU* allele. Plants that were heterozygous for the *eru* T-DNA insertion were allowed to self-pollinate and their progeny was screened toward the presence of the *eru* short RH phenotype. In addition, to gain insight into the distribution of mutant seeds along the silique, each silique was cut in half and both parts were examined separately. A smaller proportion of *eru* homozygotes in the bottom part of a silique would indicate slower growth of mutant PTs *in vivo* (Schiefelbein et al., 1993). We observed a strong deviation from the expected Mendelian inheritance ratio (25% *eru*^HOM^_,_ 50% *eru*^HET^, and 25% WT) and found consistently more seedlings with a normal RH phenotype (86.1 ± 1.4% *eru*^HET^+ WT), and thus less homozygous *eru* progeny (13.9 ± 1.4% *eru*^HOM^) in both silique halves (**Figure 4D**), illustrating decreased fertilization efficiency of *eru* pollen. We observed no difference in inheritance ratio between the top and bottom silique parts. Given the complete lack of ovular *ERU* transcription (shown by public transcriptomics data and *proERU::GUS* expression), the altered *eru* transmission efficiency is ought to be specifically due to a PT defect (**Figures 1A,B**).

Together, these data show that ERU regulates PT targeting *in vivo* and is an important component of the fertilization pathway in *Arabidopsis thaliana*.

## Discussion

The PT and RH tip growth pathways likely share multiple regulatory components (Schiefelbein et al., 1993; Procissi et al., 2003; Becker et al., 2014; Schoenaers et al., 2017). The CrRLK1L family protein ERULUS was shown to have a crucial role in regulating RH morphogenesis in *Arabidopsis thaliana* (Bai et al., 2014a; Haruta et al., 2014). In agreement with public transcriptomics data (**Figure 1A**; Pina et al., 2005; Wang et al., 2008) we now found that *ERU* is also transcribed in mature pollen grains and growing PTs (**Figure 1B**). The *ERU* promoter contains several pollen-specific cis-elements (Supplementary Table 1) and, by GUS-staining of stably transformed WT x *proERU::GUS* lines, we showed that the promoter is highly active throughout PT development (**Figure 1B**). *ERU* transcription was not detected in any other flower tissues. Taken together, these data suggest that *ERU* functions specifically in tip growing cells.

Our promoter analysis also identified two canonical auxin response elements (AuxREs), which suggests auxin-regulated *ERU* transcription (Supplementary Table 1). Auxins are known to play a determining role in pollen development and PT growth (Pina et al., 2005; Cheng et al., 2006; Wu et al., 2008; Dal Bosco et al., 2012; Yang et al., 2013). Auxin regulates transcription through the action of ARF transcription factors. The latter bind directly to the consensus auxin response element TGTCTC (Ulmasov et al., 1997). Several ARFs are expressed in dry and germinated pollen (Supplementary Figure 3), suggesting that *ERU* transcription in pollen could be regulated by auxin through present ARFs binding to its promoter.

In RHs, loss of *ERU* function results in early growth cessation and RH bulging. As such, ERU was shown to be involved in regulating the tip growth stage of RH development. Our data now shows that ERU is also involved in regulating PT tip growth in response to the [Ca^2+^]_ext_. When grown on low [Ca^2+^]_ext_, *eru* PTs grew slower than WT PTs, but displayed a normal morphology. In addition, *in vivo* PT growth occurred normally regarding morphology up to the ovule targeting stage (see further). This relatively subtle growth phenotype stands in stark contrast to the *eru* RH phenotype. However, despite that fact that we do not know what is causing the *eru* PT growth defect, and the similarities between PT and RH tip growth, important differences between both apical growth regimes exist and could relate to the observed phenotypic difference (Schoenaers et al., 2017). To better understand the mechanism(s) related to affected *eru* PT growth we studied [Ca^2+^]_cyt_ dynamics under different [Ca^2+^]_ext_.

At the apex of growing PTs, hyperpolarization activated calcium channels import calcium from the extracellular region (Véry and Davies, 2000; Qu et al., 2007) leading to a steep calcium gradient at the growing tip. Similar to RHs, the existence of such an oscillating tip-focused calcium gradient is essential to regulate PT growth speed and growth directionality (Bibikova et al., 1997; Holdaway-Clarke et al., 1997; Michard et al., 2011). Our results now show that both lower and higher than control [Ca^2+^]_ext_ stimulated WT PT growth, whereas a different response was observed for *eru* PTs. Importantly, we found that the *eru* mutation affects the [Ca^2+^]_cyt_ oscillations in response to [Ca^2+^]_ext_ (**Figure 3**). When grown on low [Ca^2+^] medium, *eru* pollen grew slower than WT pollen (**Figure 2**) and exhibited regular yet lower frequency [Ca^2+^]_cyt_ oscillations (**Figure 3**). The link between [Ca^2+^]_ext_, [Ca^2+^]_cyt_ and PT growth is far from fully understood and as such we cannot provide a causal explanation for the observed differences. Whether ERU is directly or indirectly involved in the regulation of [Ca^2+^]_cyt_ oscillations in growing PTs remains to be investigated. *ERU* loss-of-function roots grow short and stunted RHs which fail to accumulate apical cytoplasmic Ca^2+^. The latter is presumed to be a secondary defect due to a misbalance in ammonium transport across the tonoplast (Bai et al., 2014a). It’s important to consider that Ca^2+^-ions intersect with the tip growth pathway at several levels (Schoenaers et al., 2017). For instance, amongst a myriad of other responses, Ca^2+^ regulates cell wall plasticity through apoplastic pectin cross-linking and cytoplasmic activation of apoplastic ROS production (Rounds et al., 2011; Kaya et al., 2014). ERU has been suggested to regulate ROS production in RHs and members of the CrRLK1L family were suggested before to have a role in cell wall sensing (Lindner et al., 2012; Bai et al., 2014b; Nissen et al., 2016). As such, it would be interesting to investigate the effect of the [Ca^2+^]_ext_ on ROS production and the cell wall in *eru* PTs.

Nevertheless, earlier findings suggested that [Ca^2+^]_ext_ might be an important factor controling *in vivo* PT growth. Apoplastic Ca^2+^ levels (in Lily) and *ACA13* transcription (coding for an *Arabidopsis* plasma membrane localized Ca^2+^-ATPase which exports Ca^2+^ into the apoplast) both increase in the transmitting tract upon pollination (Zhao et al., 2004; Iwano et al., 2014). Moreover, Ca^2+^-signaling/crosstalk is of central importance for PT guidance toward the ovule and the steps preceding successful fertilization (Iwano et al., 2012; Ngo et al., 2014). Given the role of ERU in PT tip growth, its involvement in regulating the response to extracellular calcium, and the importance of controlled PT growth in plant fertilization, we investigated whether *eru* PTs targeted the ovules and fertilized the eggs with an efficiency similar to that of WT PTs. Fertility strongly depends on successful directional tip growth and directional cues provided by the female tissue (Higashiyama and Takeuchi, 2015). Therefore PTs possess highly conserved and specific mechanisms to sense and integrate responses to their extracellular environment (Higashiyama and Takeuchi, 2015). Our results show that, *in vivo*, *eru* pollen are less competitive than WT pollen in terms of plant fertilization efficiency (**Figure 4D**). More so, we found that *eru* PTs grown through WT tissue show aberrant ovule targeting (**Figures 4A,B**). Hence, proportionally more mutant PTs grew around the funiculus or made multiple turns before targeting the micropyle (**Figure 4A**). The *eru* PT targeting and fertilization phenotypes are strikingly similar to what has been observed for the double *lip1 lip2* mutant, which is defective in two PT plasma membrane localized RLKs (Liu et al., 2013). LIP1 and LIP2 are thought to be crucial components of the receptor complex regulating PT guidance in response to the micropyle-secreted AtLURE1 signaling peptide. Since both proteins are cytoplasmic, the LURE receptor remains to be identified. It is tempting to hypothesize that ERU could function as this receptor, and as such could regulate PT targeting in a complex with LIP1 and LIP2.

In the female synergids, a similar mechanism seems to exist. FER and LRE regulate PT attraction and PT/synergid crosstalk by perception of a yet to identify PT-derived ligand (Ngo et al., 2014; Liu et al., 2016). FER is thought to be the receptor for this ligand, whereas the cytoplasm localized, membrane-anchored EF-hand containing LRE has been hypothesized to form a complex with FER and regulate ligand-induced Ca^2+^ crosstalk between the PT and synergids.

Contrastingly, PTs targeted to *fer* (-/-) and *lre* (-/-) ovules show PT overgrowth inside the female tissue due to failed PT rupture rather than mistargeting prior to physical male-female contact (Huck et al., 2003; Liu et al., 2016). The latter suggests that FER- and ERU-mediated guidance are spatiotemporally separated events. As to investigate this presumption, it would be crucial to investigate PT targeting in double *eru/fer* mutant lines. This could lead to a better understanding of the different stages of PT/ovule cross-talk, and provide excellent knowledge on the signal pathways involved.

We identified ERU as an important component of the fertilization process in *Arabidopsis*. How ERU regulates *in vivo* PT targeting remains to be investigated. However, based on the common role of ERU in regulating [Ca^2+^]_cyt_ dynamics in both RHs and PTs, the ubiquity of calcium signaling throughout the PT growth and fertilization pathway, and the Ca^2+^-associated role of other CrRLK1Ls during PT development, it is sensible to hypothesize that altered Ca^2+^ signaling in *eru* PTs could be an important aspect relating to the observed *in vivo* phenotype. Nevertheless, characterization of the direct mechanistic involvement of ERU in the PT targeting process is a key element to be addressed.

## Author Contributions

SS and DB identified and phenotyped the mutant. SS and DB performed microscopy and gene expression studies. SS and AC performed calcium measurements. SS and DB analyzed the data and KV wrote the article with contribution from SS, DB, and AC. SS, DB, AC, and KV conceived the project and KV supervised the research.

## Conflict of Interest Statement

The authors declare that the research was conducted in the absence of any commercial or financial relationships that could be construed as a potential conflict of interest. The reviewer MH and handling Editor declared their shared affiliation.

## Footnotes

1 http://www.dna.affrc.go.jp/PLACE/

2 http://www.lifetechnologies.com/
