# Supplementary material for: The Kinase ERULUS Controls Pollen Tube Targeting and Growth in Arabidopsis thaliana
Source: Front Plant Sci. 2017 Nov 14;8:1942. doi: 10.3389/fpls.2017.01942 (PMC5694544; doi:10.3389/fpls.2017.01942)
Supplement: Supplementary file 1 [file Supplementary_Figures_and_Tables.pdf]

Supplementary Material

The kinase ERULUS controls pollen tube targeting and growth in *Arabidopsis thaliana*.

Sébastjen Schoenaers, Daria Balcerowicz, Alex Costa, Kris Vissenberg*

*** Correspondence:** Kris Vissenberg: kris.vissenberg@uantwerpen.be

1 Supplementary Figures and Tables

1.1 Supplementary Figures

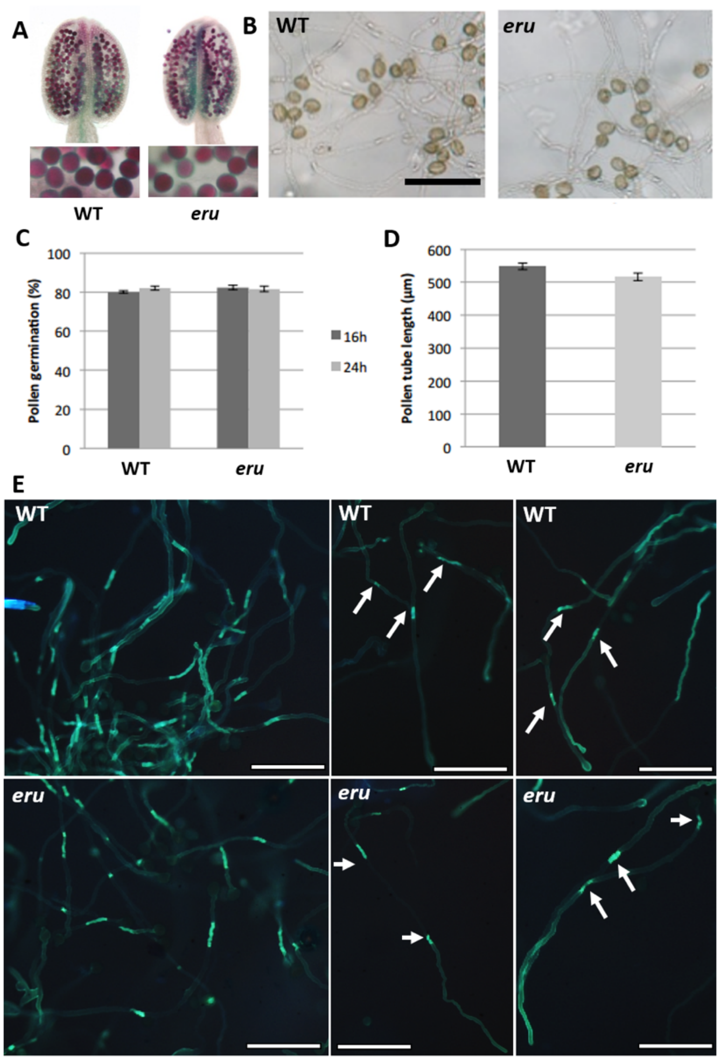

Supplementary Figure 1. Phenotyping of WT and *eru* pollen and PTs.

(A) Alexander staining of mature WT and *eru* pollen. (B) Morphology of *in vitro* germinated WT and *eru* PTs. (C) *in vitro* pollen germination percentage of WT and *eru* pollen at 16 and 24h after pollination. (D) Final PT length of *in vitro* germinated WT and *eru* PTs. (E) Callose plug formation in *in vitro* grown WT and *eru* PTs. Arrows indicate DAB-stained callose plugs in fully grown PTs. Scale bars = 100 µm. Error bars indicate SE. Significance codes p-value: 0.05 '*' 0.01 '**' <0.001 '***'.

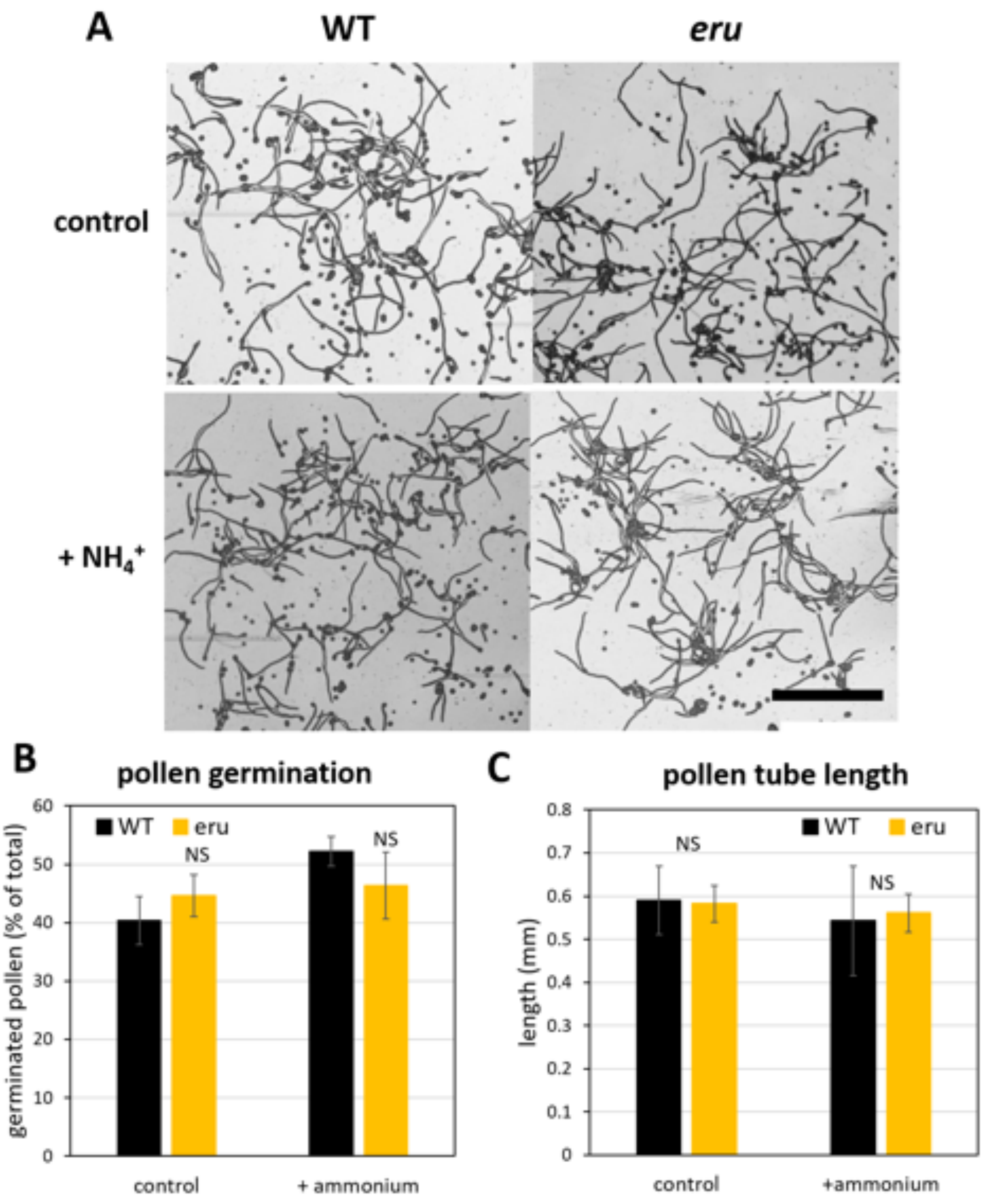

Supplementary Figure 2. Phenotyping of WT and *eru* PTs upon ammonium supplementation.

(A) Representative images of *in vitro* germinated WT and *eru* PTs under control (top) and ammonium supplemented (bottom) conditions. Scale bar = 500 µm. (B) pollen germination percentage of WT and *eru* pollen under control and ammonium supplemented conditions. (C) PT length of WT and *eru* PTs 2.5 h after *in vitro* pollination. Error bars represent SE. Significance codes p-value: 0.05 '*' 0.01 '**' <0.001 '***'.

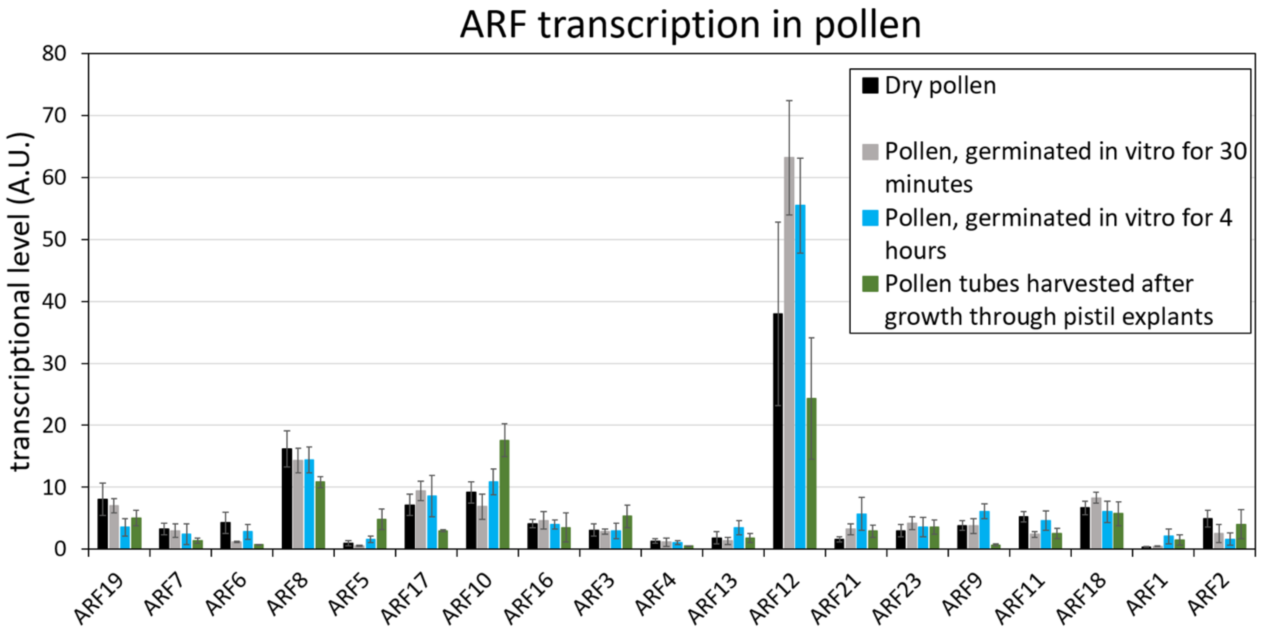

Supplementary Figure 3. Pollen- and PT-specific transcription of Auxin Response Factors (ARFs).

Bar plot showing the transcription level of individual ARFs in pollen and PTs as measured in four publicly available transcriptomics datasets.

1.2 Supplementary tables

SI table 1: Regulatory cis-elements found in the 1500 bp *ERU* promoter region

Cis-element	Sequence	Position upstream from ATG/Strand	Description
RHERPATEXPA7	KCACGW	400 (+)<br>417 (-)	"Right part of RHEs (Root Hair-specific cis-Elements)" conserved among the Arabidopsis thaliana A7 (AtEXPA7) orthologous (and paralogous) genes from diverse angiosperm species with different hair distribution patterns; K=G/T; W=T/A
POLLEN1LELAT52	AGAAA	309 (+)<br>371 (+)<br>107 (-)<br>186 (-)<br>282 (-)<br>386 (-)	One of two co-dependent regulatory elements responsible for pollen specific activation of tomato lat52 gene; AGAAA and TCCACCATA are required for pollen specific expression; Also found in the promoter of tomato endo-beta-mannanase gene (LeMAN5)
QELEMENTZMZM13	AGGTCA	9(+)	"Q(quantitative)-element" in maize ZM13 gene promoter; Involved in expression enhancing activity; ZM13 is a pollen-specific maize homolog of tomato LAT52 gene

Supplementary Table 1. ***ERU*** **promoter analysis**

Regulatory cis-elements identified in the 1500 bp *ERU* promoter region.
